# Survey datasets on the externalizing behaviors of primary school pupils and secondary school students in some selected schools in Ogun State, Nigeria

**DOI:** 10.1016/j.dib.2017.06.025

**Published:** 2017-06-16

**Authors:** Sheila A. Bishop, Enahoro A. Owoloko, Hilary I. Okagbue, Pelumi E. Oguntunde, Oluwole A. Odetunmibi, Abiodun A. Opanuga

**Affiliations:** Department of Mathematics, Covenant University, Canaanland, Ota, Nigeria

**Keywords:** Externalizing behavior, Achenbach manual, Survey, Questionnaire

## Abstract

This data article contains the partial analysis (descriptive statistics) of data obtained from 1770 primary school pupils and secondary school students in three Local Government Areas of Ogun State, Nigeria. The schools are either privately owned or public (government owned) schools. The aim of the field survey is to measure the level and patterns of externalizing behavior of the respondents. The data was collected using a standardized questionnaire. The questionnaire is a modification of Achenbach manual for Child behavior checklist (Achenbach, 2001) [1] and manual for Youth self-report (Achenbach and Rescorla, 2001) [2]. The questionnaire was designed to suit the demographic and socio-cultural nature of the target population. Analysis of the data can provide useful insights to the patterns of externalizing behavior of primary school pupils and secondary school students.

**Specifications Table**

**Table:** 

Subject area	Social Sciences
More specific subject area	Quantitative Psychology
Type of data	Table and text file
How data was acquired	Field Survey
Data format	Raw, partial analyzed (Descriptive statistics)
Experimental factors	Simple random sampling of some selected primary and secondary schools in three local Government Areas in Ogun State, Nigeria. Non response observations have been removed.
Experimental features	Sample selection of the responses of pupils and students from structured Questionnaires designed to measure their level of externalizing behavior
Data source location	Covenant University Mathematics Laboratory, Ota, Nigeria
Data accessibility	All the data are in this data article

**Value of the data**

• The data provide the descriptive statistics for the selected samples which gave an exploratory trend of the observed characteristics.
• The data when completely analyzed can provide insight on the similarities and differences in patterns of externalizing behaviors of primary school pupils and secondary school students.
• Researchers can gain more insight on the instrument of data collection, which can be adapted or adopted to suit the studied socioeconomic, demographic, psychographic and behavioral characteristics.
• The questionnaire can be used for the study of the externalizing behavior of children and early adolescent youths.
• The questionnaire can be adapted or adopted to include cohort and/or longitudinal studies.
• The data could be useful in the following research areas: child behavior, adolescent health, early child education, guidance and counseling, mental health, psychiatrics, psychopathology, Developmental psychology, Multivariate Behavioral Research, Clinical Psychology and so on. The central theme is the study of externalizing behavior instincts and observed patterns between primary school pupils and secondary school students.
• Most vulnerable groups obtained from data analysis can be singled out for counseling and monitoring by the concerned authorities thereby improving on the public health of the people.

## 1 Data

The data in this article is the set of responses solicited from 1770 primary school pupils and secondary school students in three Local Government Areas in Ogun State Nigeria. The details of the sample size are shown in Table 1a, Table 1b, Table 1c, Table 1d, Table 1e and 1f. The data was collected by the use of questionnaire. The questionnaire is a modification of Achenbach manual for child behavior checklist [1] and manual for youth self-report [2]. The nature and usefulness of the data entails that it can be analyzed using the following statistical techniques: regression analysis (ordinary least square), analysis of variance, Poisson regression, logistic models, path analysis models, latent growth curve analysis, middle level growth models, factor analysis, principal component analysis, multiple correspondence analysis, structural equation modeling, multivariate regression models, cluster analysis and so on.

**Table 1a:** School type of respondents.

**Type**	**Public**	**Private**

Number of respondents	1136	634

**Table 1b:** Educational level of respondents.

**Level**	**Primary**	**Secondary**

	368	1402

**Table 1c:** Gender of respondents.

**Gender**	**Female**	**Male**	

Number	996	774

**Table 1d:** Age of respondents.

**Age**	**Below 10**	**11–15**	**16–20**

Frequency	156	1202	412

**Table 1e:** Crosstabulation of gender and school type of respondents.

		**School**		**Total**
		**Public**	**Private**	
**Gender**	**Male**	501	273	774
	**Female**	635	361	996
**Total**		1136	634	1770

**Table 1 f:** Crosstabulation of gender and educational level of respondents.

		**Level**		**Total**
		**Primary**	**Secondary**	
**Gender**	**Male**	181	593	774
	**Female**	187	809	996
**Total**		368	1402	1770

The contents of the data are variables that determine the externalizing behavior of the respondents. These variables are as a result of under control of emotions as listed in the questionnaire. The analysis of the data can reveal the externalizing behavior of the respondents which can manifest as aggression, delinquency and hyperactivity. Furthermore, the gender, age and educational level differences in the distribution of externalizing behavior patterns can be obtained from the analysis of the data. In addition, research questions can be posed and statistical hypothesis can be tested based on the data. Finally, the data contains some variables which have not been considered in the analysis of externalizing behavior in children and adolescents and the questionnaire can serve as a benchmark tool for behavioral analysis especially in the sub Saharan region of Africa.

The data can be assessed as Supplementary data 1 and the Questionnaire can be assessed as Supplementary data 2.

### 1.1 The summary statistics of the total score of the samples

The summary statistics of the total score of the respondents is given in Table 2.

**Table 2:** Summary statistics of the total scores (the measure of the externalizing behavior).

Statistic	Value
Mean	77.75 (0.68)
Median	76
Mode	72
Standard Deviation	28.604
Variance	818.164
Skewness	0.227(0.058)
Kurtosis	-0.313(0.116)
Range	163
Minimum	5
Maximum	168
Sum	137,610
Percentile 25	57
50	76
75	97

**Fig. 1:**
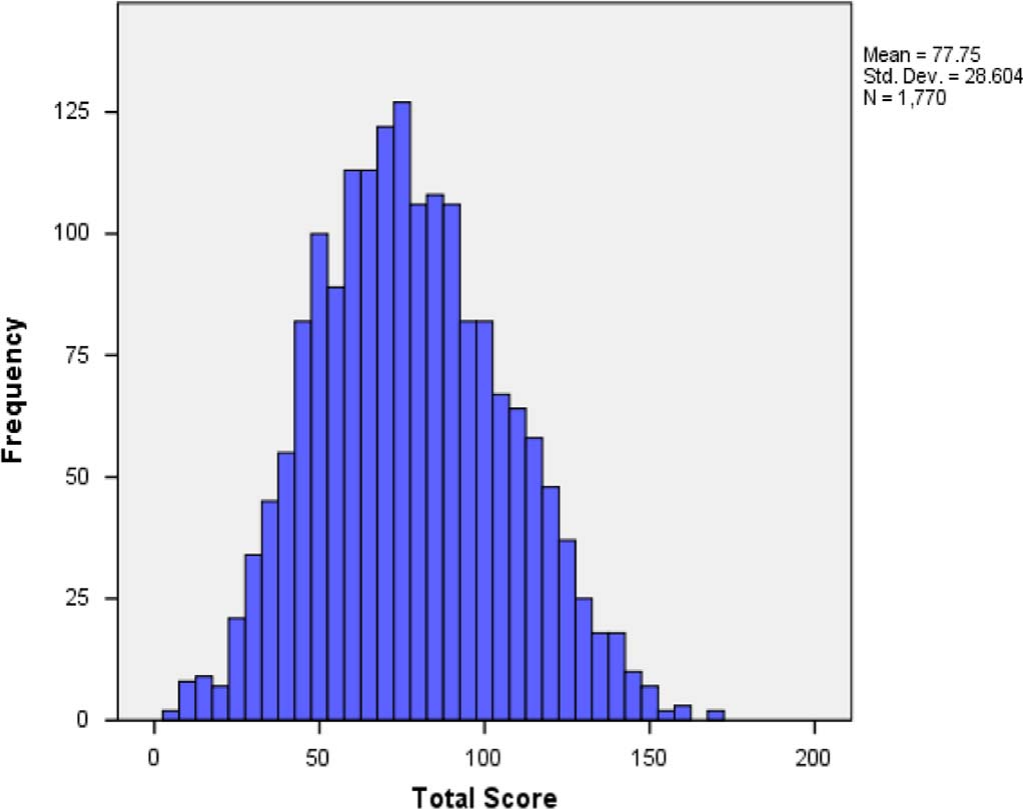
The histogram showing the total scores (the measure of the externalizing behavior).

The summary statistics was represented by a histogram shown in Fig. 1.

The histogram is the chart representation of the descriptive statistics. The histogram revealed the presence of fewer outliers (extreme behavioral patterns).

### 1.2 The percentage of the individual score compared with the total score

The percentage of the individual score compared with the total score can be computed using the formula;

(1) $$\%\;score=\frac{Individual\;score}{Total\;score}\times 100$$

The total score is 200. The summary statistics for this subsection is shown in Table 3.

**Table 3:** Summary statistics of the percentage total scores (the measure of the individual externalizing behavior compared with the Total score).

Statistic	Value
Mean	38.873 (0.3399)
Median	38
Mode	36
Standard Deviation	14.3018
Variance	204.541
Skewness	0.227(0.058)
Kurtosis	-0.313(0.116)
Range	81.5
Minimum	2.5
Maximum	84
Sum	68,805
Percentile 25	28.5
50	38
75	48.5

### 1.3 Gender Differences in the distribution of the externalizing behavior among the respondents

The summary statistics for the gender differences in the distribution of the total score for the primary school pupils and secondary school students is shown in Table 4.

**Table 4:** Summary statistics of the gender differences in the measure of the externalizing behavior of the respondents.

Statistic	Male	Female
Mean	79.56 (1.079)	76.34 (0.868)
Median	78.5	74
Standard Deviation	30.011	27.392
Variance	900.653	750.348
Skewness	0.227(0.088)	0.197(0.077)
Kurtosis	−0.314(0.176)	−0.379(0.155)
Range	163	151
Minimum	5	5
Maximum	168	156

### 1.4 School differences in the distribution of the externalizing behavior among the respondents

The summary statistics for the school differences in the distribution of the total score for the primary school pupils and secondary school students is shown in Table 5.

**Table 5:** Summary Statistics of the school differences in the measure of the externalizing behavior of the respondents.

Statistic	Public	Private
Mean	74.77 (0.838)	83.08 (1.132)
Median	72	83
Standard Deviation	28.233	28.511
Variance	797.078	812.878
Skewness	0.352(0.073)	0.014 (0.097)
Kurtosis	−0.101(0.145)	−0.459 (0.194)
Range	163	146
Minimum	5	10
Maximum	168	156

### 1.5 Age Differences in the distribution of the externalizing behavior among the respondents

The summary statistics for the age differences in the distribution of the total score for the primary school pupils and secondary school students is shown in Table 6.

**Table 6:** Summary Statistics of the age differences in the measure of the externalizing behavior of the respondents.

Statistic	Below 10 years	11–15 years	16–20 years
Mean	78.62 (2.483)	78.09 (0.817)	76.41(1.405)
Median	77	76	75.5
Standard Deviation	31.011	28.318	28.514
Variance	961.709	801.907	813.074
Skewness	0.022(0.194)	0.202 (0.071)	0.396 (0.120)
Kurtosis	-0.837 (0.386)	-0.269 (0.141)	-0.132 (0.240)
Range	132	163	154
Minimum	12	5	14
Maximum	144	168	168

### 1.6 School level Differences in the distribution of the externalizing behavior among the respondents

The summary statistics for the school level differences in the distribution of the total score for the primary school pupils and secondary school students is shown in Table 7.

**Table 7:** Summary statistics of the school level differences in the measure of the externalizing behavior of the respondents.

Statistic	Primary	Secondary
Mean	79.99 (1.671)	77.16 (0.737)
Median	81	75
Standard Deviation	32.054	27.609
Variance	1027.428	762.261
Skewness	0.004 (0.127)	0.296 (0.065)
Kurtosis	−0.681 (0.254)	−0.181 (0.131)
Range	151	163
Minimum	8	5
Maximum	159	168

### 1.7 The mean and standard deviation of all the questions in the questionnaire

The mean and standard deviation of all the questions in the questionnaire responded by the primary school pupils and secondary school students is shown in Table 8.

**Table 8:** The mean and standard deviation of all questions obtained from the respondents.

Question	Mean	S.D.	Question	Mean	S.D.	Question	Mean	S.D.	Question	Mean	S.D.
1	1.35	0.692	26	0.57	0.751	51	0.87	0.861	76	0.19	0.520
2	1.04	0.811	27	0.81	0.743	52	1.17	0.862	77	0.85	0.840
3	0.54	0.789	28	1.05	0.829	53	0.89	0.853	78	0.90	0.812
4	0.23	0.565	29	0.90	0.875	54	1.08	0.834	79	0.45	0.694
5	0.65	0.774	30	0.99	0.818	55	0.34	0.646	80	0.23	0.570
6	0.56	0.767	31	0.59	0.771	56	0.31	0.642	81	0.84	0.827
7	1.01	0.830	32	0.48	0.738	57	1.11	0.860	82	0.88	0.846
8	0.90	0.845	33	1.49	0.738	58	1.36	0.809	83	0.66	0.757
9	1.27	0.690	34	0.85	0.827	59	1.02	0.808	84	0.39	0.692
10	0.46	0.681	35	1.39	0.770	60	1.05	0.826	85	0.88	0.832
11	0.20	0.494	36	1.05	0.829	61	0.60	0.786	86	0.57	0.779
12	0.18	0.484	37	0.63	0.826	62	0.94	0.816	87	0.95	0.818
13	0.40	0.676	38	0.77	0.869	63	0.40	0.671	88	0.96	0.807
14	0.54	0.750	39	0.83	0.877	64	0.70	0.809	89	1.26	0.830
15	1.05	0.838	40	0.99	0.823	65	1.21	0.825	90	1.16	0.848
16	0.69	0.790	41	1.45	0.779	66	0/79	0.802	91	1.00	0.813
17	0.75	0.787	42	0.38	0.654	67	0.42	0.725	92	0.66	0.770
18	0.57	0.714	43	0.51	0.727	68	0.50	0.721	93	0.54	0.705
19	1.54	0.683	44	0.46	0.731	69	0.89	0.834	94	1.15	0.792
20	1.06	0.811	45	0.51	0.748	70	1.28	0.816	95	0.94	0.812
21	0.76	0.779	46	0.22	0.557	71	0.63	0.794	96	0.61	0.764
22	0.60	0.811	47	0.88	0.813	72	0.35	0.671	97	0.61	0.739
23	0.61	0.748	48	0.78	0.808	73	0.71	0.770	98	0.38	0.667
24	0.61	0.758	49	0.96	0.821	74	0.69	0.789	99	0.31	0.624
25	0.73	0.755	50	1.51	0.732	75	0.87	0.801	100	0.84	0.789

### 1.8 The distribution of the responses from the questions

The distribution of the responses from all the questions contained in the questionnaire is shown in Table 9.

**Table 9:** The overall distribution of the responses from the respondents.

Question	Not True	Somewhat True	Often True	Question	Not True	Somewhat True	Often True
1	222	701	847	51	787	427	556
2	545	603	622	52	535	406	829
3	1139	300	331	53	756	461	553
4	1498	144	128	54	553	531	686
5	955	488	327	55	1337	263	170
6	1082	386	302	56	1396	199	175
7	602	552	616	57	571	441	758
8	726	490	554	58	374	378	1018
9	434	417	919	59	560	614	596
10	1140	440	190	60	559	558	653
11	1498	196	76	61	1047	390	333
12	1531	162	77	62	641	587	542
13	1256	322	192	63	1253	331	186
14	1095	397	278	64	923	453	394
15	580	523	667	65	454	488	828
16	913	495	362	66	793	554	423
17	829	561	380	67	1282	238	250
18	1002	534	234	68	1121	411	238
19	192	437	1141	69	722	518	530
20	534	601	635	70	410	452	908
21	799	596	375	71	1004	414	352
22	1083	318	369	72	1338	236	196
23	968	517	285	73	859	569	342
24	982	489	299	74	914	496	360
25	801	638	331	75	698	604	468
26	1042	446	282	76	1536	131	103
27	689	730	351	77	772	485	513
28	567	550	653	78	680	585	505
29	778	398	594	79	1171	393	206
30	601	585	584	80	1497	142	131
31	1031	427	312	81	771	514	485
32	1174	335	261	82	756	478	536
33	261	374	1135	83	905	556	309
34	757	521	492	84	1294	263	213
35	314	449	1007	85	733	519	518
36	564	551	655	86	1088	362	320
37	1051	321	398	87	642	582	546
38	921	339	510	88	610	615	545
39	861	355	554	89	440	432	898
40	603	573	594	90	517	454	799
41	317	334	1119	91	584	600	586
42	1273	327	170	92	921	522	327
43	1114	410	246	93	1037	513	220
44	1215	302	253	94	440	620	710
45	1144	352	274	95	640	597	533
46	1497	152	121	96	990	474	306
47	699	578	493	97	970	529	271
48	810	533	427	98	1284	301	185
49	630	576	564	99	1384	230	156
50	253	356	1161	100	713	625	432

## 2 Experimental design, materials and methods

Researches on externalizing behavior and other related fields are often conducted by the use of standardized questionnaires. Details on other research aimed at studying the nature, causes, distribution and management of externalizing behavior in children and adolescents can be found in [3], [4], [5], [6], [7], [8], [9], [10], [11], [12], [13], [14], [15], [16], [17], [18], [19], [20], [21], [22], [23], [24], [25], [26], [27], [28], [29], [30]. Sample (field) survey was used to obtain the data, similar researches that used field survey to obtain their data can also be found in [31], [32], [33], [34], [35], [36], [37], [38], [39], [40], [41], [42], [43], [44], [45].

Simple random sampling (SRS) was used to obtain the data across the three Local Government areas (LGA) in Ogun State, Nigeria. The selected LGAs are Ado-Odo/Ota, Ifo and Yewa South, which are in close proximity to each other. The choice of the target population reflects the views of both the urban and rural respondents, reflecting the demographics in the State. The focus is on the gender, age, school type and educational level of the distribution of the externalizing behavior patterns of the respondents.

The differences between the rural and urban externalizing behavior pattern is open for further research. The questionnaire was given to pupils and students of public/private primary and secondary schools. The sampling was solely on without replacement and the non-response was excluded from the final data. Non responses are categorized as incomplete data as a result of partial or no responses from the respondents. Inclusion of such data can be detrimental to the estimation of the population parameters.

The internal consistencies and the reliability of scale for the questions Q1–Q100 in the questionnaire is shown in Table 10. The table showed a high random nature of the data and is very reliable for statistical analysis.

**Table 10:** Summary of the measure of reliability of the data.

Statistic	Value
Cronbach׳s alpha	0.937
Correlation between forms	0.773
Spearman Brown Coefficient	0.872
Gutman Split-Half Coefficient	0.866
Reliability of scale	0.937
Lambda 1	0.928
2	0.938
3	0.937
4	0.866
5	0.931
