# Supplementary material for: Survey datasets on the externalizing behaviors of primary school pupils and secondary school students in some selected schools in Ogun State, Nigeria
Source: Data Brief. 2017 Jun 16;13:469–79. doi: 10.1016/j.dib.2017.06.025 (PMC5487303; doi:10.1016/j.dib.2017.06.025)
Supplement: Supplementary file 3 — Supplementary material [file mmc3.pdf]

0 = Not True 1 = Somewhat or Sometimes True 2 = Very True or Often True

This questionnaire is designed by the Probability and Statistics Sub-cluster in Covenant University, Ota, Nigeria. Its specific aim is to know the externalizing behavior of students both at the secondary and primary school level. Kindly answer the questions correctly as your personal details will be made confidential. Thank you in advance.

Section 1

Kindly tick the appropriate.

School: **Public** **Private.**

Age bracket: **Below 10** **11 - 15** **16 – 20**

Gender: **Male** **Female**

Level: **Primary** **Secondary**

Section 2

0 1 2 1. I like to do things in my own ways.

0 1 2 2. I don't see the need of always reporting to my teacher when I am offended by others

0 1 2 3. I believe that my parents are often too busy to attend to my complaints.

0 1 2 4. I often run away from the house to avoid being disciplined at home.

0 1 2 5. I repeat certain acts over and over again.

0 1 2 6. I often scream.

0 1 2 7. I prefer viewing TV to sleeping at weekends.

0 1 2 8. I am easily distracted from doing my assignments in the class.

0 1 2 9. I often shout to show that I can stand up for my right.

0 1 2 10. I have to lie to avoid been disciplined.

0 1 2 11. I steal when I am hungry at home.

0 1 2 12. I believe it is OK to take other peoples things especially when i need them most.

0 1 2 13. I am always tempted to use my offerings to buy snacks.

0 1 2 14. I use force on people that don't understand me.

0 1 2 15. I cannot control my temper when I am angry that's why I don't like people hurting me.

0 1 2 16. I laugh at people that don't behave well.

0 1 2 17. I prefer to spend more time than usual at break.

0 1 2 18. I always see my classes as boring.

0 1 2 19. I am always worried when things are not going the way I want them.

0 1 2 20. I cannot sit at a place for so long.

0 1 2 21. I shout at my younger ones.

0 1 2 22. I often use my parents' phones to call my friends.

0 1 2 23. I often practice what I saw in the TV.

0 1 2 24. I can push somebody that blocked my way.

0 1 2 25. I prefer to listen to my friends because they are always right.

0 1 2 26. I prefer to be with the opposite sex.

0 1 2 27. I often get injured while playing.

0 1 2 28. I have fought to defend myself.

0 1 2 29. I don't like been disciplined at school.

0 1 2 30. I always want people to know my own view even if it means talking back at them.

0 1 2 31. I don't forgive people easily.

0 = Not True 1 = Somewhat or Sometimes True 2 = Very True or Often True

0 1 2 32. I don't like doing homework at the weekends.

0 1 2 33. I don't like to be disturbed while sleeping.

0 1 2 34. I don't like people visiting our house too often.

0 1 2 35. I always argue when I feel cheated on.

0 1 2 36. I always argue with people that are disrespectful to me.

0 1 2 37. I hate pets.

0 1 2 38. I see myself as being more important than others.

0 1 2 39. I see obeying traffic rules as boring especially when there are no incoming cars or motorcycles.

0 1 2 40. I find it hard to wait and stay in a queue.

0 1 2 41. I cannot tolerate people cursing at me.

0 1 2 42. I prefer been at home than in school.

0 1 2 43. I think that it's not always OK for me to obey the rules at all times.

0 1 2 44. I don't like waking up very early for school.

0 1 2 45. I often prefer to be in my friend's class.

0 1 2 46. I would like to taste alcohol when I grow older.

0 1 2 47. I can hardly hold things in my hands for long without dropping them.

0 1 2 48. I attack people when they think that I am weak and without power to react.

0 1 2 49. I often fight back when I am beaten by another.

0 1 2 50. I must always defend myself.

0 1 2 51. I often draw and write on the walls to show my knowledge of Arts and English.

0 1 2 52. I can turn on TV set when my parents are not around.

0 1 2 53. I can slap when I am angry.

0 1 2 54. I can slap back when slapped.

0 1 2 55. I feel like shouting at my teacher at times.

0 1 2 56. I often don't like washing my school uniforms.

0 1 2 57. I have pushed someone before.

0 1 2 58. I can always cross the main roads without any assistance.

0 1 2 59. I don't like people looking at me.

0 1 2 60. I can help my friends during test or exam.

0 1 2 61. I often don't like to go home early, to avoid doing some house chores.

0 1 2 62. I believe that those that I fought with, deserve it.

0 1 2 63. I can steal only small things at home.

0 1 2 64. I don't feel like following the rules as long as I am right.

0 1 2 65. I often speak loud when I am angry.

0 1 2 66. I often prefer to stay outside my house.

0 1 2 67. I don't see the reason of always thanking people when they do something to me.

0 1 2 68. I don't like helping out with house chores.

0 1 2 69. I hate to be disturbed while playing.

0 1 2 70. I cannot tolerate being insulted by friends, neighbors or peers.

0 1 2 71. I always like to see people that hurt me cry.

0 1 2 72. I can take other people's things when I didn't see mine.

0 = Not True 1 = Somewhat or Sometimes True 2 = Very True or Often True

0 1 2 73. I can talk to people even when the class is going on.

0 1 2 74. I can eat my siblings' food when 1 am hungry.

0 1 2 75. I love to move around the classes to know what is happening there.

0 1 2 76. I often don't brush my teeth.

0 1 2 77. I can throw stone at dogs, cats.

0 1 2 78. I don't like staying indoors.

0 1 2 79. I don't like staying in my seat in the class.

0 1 2 80. I watch people when they are bathing.

0 1 2 81. I prefer to be addressed by my nickname.

0 1 2 82. I love to give people nick names.

0 1 2 83. I often fell while running.

0 1 2 84. I can frame sick to avoid going to school.

0 1 2 85. I like to play music very loud or listen to loud music.

0 1 2 86. I can use my reading desk to play music.

0 1 2 87. I can laugh very loud when amused.

0 1 2 88. I watch people when they are quarreling.

0 1 2 89. I can handle any phone without being taught.

0 1 2 90. I can operate most of the electrical appliances in the house.

0 1 2 91. I often jump and shout in class whenever 1 am very happy.

0 1 2 92. I often laugh at people that are not properly dressed.

0 1 2 93. I found it hard to do house chores.

0 1 2 94. I often complain to show my feelings.

0 1 2 95. I believe that my parents cannot understand why I do some certain things.

0 1 2 96. I found it hard to apologize.

0 1 2 97. I often misplace keys.

0 1 2 98. I often search my sibling's bags/ friends' school bags.

0 1 2 99. I often don't like wearing my school uniform to school.

0 1 2 100. I cannot tell my parents when I spoil things in the house.
